# Impact of Frailty on Hippocampal Volume in Patients with Chronic Obstructive Pulmonary Disease

**DOI:** 10.3390/biomedicines9091103

**Published:** 2021-08-28

**Authors:** Shun Takahashi, Tsunahiko Hirano, Kasumi Yasuda, Tomohiro Donishi, Kazuyoshi Suga, Keiko Doi, Keiji Oishi, Shuichiro Ohata, Yoriyuki Murata, Yoshikazu Yamaji, Maki Asami-Noyama, Nobutaka Edakuni, Kazuto Matsunaga

**Affiliations:** 1 Department of Neuropsychiatry, Wakayama Medical University, Wakayama 641-0012, Japan; t-shun@wakayama-med.ac.jp (S.T.); y-kasumi@wakayama-med.ac.jp (K.Y.); 2 Department of Respiratory Medicine and Infectious Disease, Graduate School of Medicine, Yamaguchi University, Ube 755-8505, Japan; decem119@yamaguchi-u.ac.jp (K.D.); j015ebponyou@gmail.com (S.O.); yyamaji@yamaguchi-u.ac.jp (Y.Y.); noyamama@yamaguchi-u.ac.jp (M.A.-N.); edakuni@yamaguchi-u.ac.jp (N.E.); kazmatsu@yamaguchi-u.ac.jp (K.M.); 3 Department of System Neurophysiology, Wakayama Medical University, Wakayama 641-0012, Japan; tdonishi@wakayama-med.ac.jp; 4 Department of Radiology, St. Hill Hospital, Ube 755-0155, Japan; sugar@sthill-hp.or.jp; 5 Department of Medicine and Clinical Science, Graduate School of Medicine, Yamaguchi University, Ube 755-8505, Japan; ohishk@yamaguchi-u.ac.jp (K.O.); ymurata-ygc@umin.ac.jp (Y.M.)

**Keywords:** chronic obstructive pulmonary disease, hippocampus, frailty, depression, QOL

## Abstract

Brain frailty may be related to the pathophysiology of poor clinical outcomes in chronic obstructive pulmonary disease (COPD). This study examines the relationship between hippocampal subfield volumes and frailty and depressive symptoms, and their combined association with quality of life (QOL) in patients with COPD. The study involved 40 patients with COPD. Frailty, depressive symptoms and QOL were assessed using Kihon Checklist (KCL), Hospital Anxiety and Depression Scale (HADS), and World Health Organization Quality of Life Assessment (WHO/QOL-26). Anatomical MRI data were acquired, and volumes of the hippocampal subfields were obtained using FreeSurfer (version 6.0). Statistically, HADS score had significant association with WHO/QOL-26 and KCL scores. KCL scores were significantly associated with volumes of left and right whole hippocampi, presubiculum and subiculum, but HADS score had no significant association with whole hippocampi or hippocampal subfield volumes. Meanwhile, WHO/QOL-26 score was significantly associated with volume of the left CA1. There was a significant association between frailty, depression, and QOL. Hippocampal pathology was related to frailty and, to some extent, with QOL in patients with COPD. Our results suggest the impact of frailty on hippocampal volume and their combined associations with poor QOL in COPD.

## 1. Introduction

Chronic obstructive pulmonary disease (COPD) is a highly prevalent chronic disease, the progressive course of which impacts upon mortality and disturbs various aspects of the patient’s life [1,2]. Chronic systemic inflammatory reaction causes extra-pulmonary manifestation in COPD, such as motor and psychiatric symptoms [3,4,5,6,7]. Systematic review and meta-analyses have reported the prevalence of frailty and depression in patients with COPD as 19% [8] and 27.1% [9], respectively.

In patients with COPD, poor clinical outcomes and a higher rate of mortality are associated with frailty [10,11,12] and co-morbid depression [13,14,15,16,17,18]. COPD has been shown in longitudinal studies to increase the risk of developing depression, although there is also an inverse relationship: depression adversely affects the prognosis of COPD [19]. Similarly, individuals with frailty have been reported to have an increased likelihood of developing depression, and vice versa [20].

Hypoxia, inflammatory mediators and cerebral small vessel disease are leading brain pathologies of patients with COPD [21,22]. The hippocampus is a plastic and vulnerable region of the brain that is especially susceptible to damage [23]. Hippocampal volume loss has been reported in patients with COPD [24,25], in frailty [26,27] and in patients with depression [28].

We hypothesized that volume loss in hippocampal subfields might be associated with poor clinical outcomes in patients with COPD. We, therefore, examined the relationship between hippocampal subfield volumes and frailty, depressive symptoms and quality of life (QOL). We also aimed to elucidate the association between frailty and depressive symptoms and QOL in patients with COPD.

## 2. Materials and Methods

### 2.1. Subjects

Forty patients with COPD diagnosed by a pulmonologist and recruited from the Yamaguchi Medical University Hospital were included in the study (Table 1). All patients were diagnosed and treated based on the Global Initiative for Chronic Obstructive Lung Disease (GOLD) guidelines (Global Initiative for Chronic Obstructive Lung Disease. 2021, available online: www.goldcopd.inc. (accessed on 10 June 2021)). They were all in a stable condition and had no exacerbations for at least three months prior to the study. We excluded patients with other pulmonary diseases, such as interstitial lung disease, and those with disorders that would prevent them from completing the study assessments. This study was approved by the ethics committees at Yamaguchi University (6 June 2016) and Wakayama Medical University (19 August 2016), and the trial was registered at UMIN-ICDR (UMIN000024645, 13 October 2016). Written informed consent was obtained from all patients.

### 2.2. Clinical Assessments

Frailty, depressive symptoms and QOL were assessed using Kihon Checklist (KCL), Hospital Anxiety and Depression Scale (HADS) and WHO/QOL-26, respectively. KCL is a self-reported comprehensive health checklist designed by a study group from the Japanese Ministry of Health, Labor and Welfare. It is used as a screening tool of frailty for community-dwelling older adults [29] and patients with COPD [30,31,32].

### 2.3. Neuroimaging Analysis

Anatomical MRI data on a 3.0T MR scanner (Achieva 3.0T Quasar Dual; Philips Medical Systems) was acquired using an 8ch SENSE head coil. A 3D fastfield echo T1-weighted sequence was used for anatomical MRI (TR/TE = 7.0/3.3 ms, FOV = 256 mm, 200 slices, acquisition voxel size = 1.00 × 1.00 × 1.00 mm, and a slice thickness = 1.0 mm). Volumes of the hippocampal subfields and estimated total intracranial volume (eTIV) in each hemisphere were obtained using FreeSurfer software (version 6.0) (https://surfer.nmr.mgh.harvard.edu, accessed on 10 June 2021) (Figure S1). In segmentation of the hippocampus, to reduce multiple comparisons, we focused on seven main subfields (CA1, CA3, CA4, parasubiculum, presubiculum, subiculum, GC-ML-DG). The quality of preprocessed images was visually checked by S.T. and K.Y.

### 2.4. Statistics

All statistical analyses were performed using IBM SPSS Statistics for Windows, Version 22 (IBM Japan, Ltd., Tokyo, Japan). Normality of data was assessed using the Shapiro–Wilk test with statistical significance set at *p* < 0.05. There were non-normal distributions of volumes of left and right GC-ML-GD, right whole hippocampus, right CA1, right CA3, right CA4, and KCL score. The other hippocampal subfield volumes, age, HADS score, WHO/QOL-26 score, and values of respiratory function (%VC, %FVC, %FEV1, %DLco/VA) had normal distribution. Subjects were grouped into high, moderate, and low QOL groups according to top and bottom 25th percentile point of WHO/QOL-26 (high QOL: 4.38–3.50, moderate QOL: 3.42–2.69, low QOL: 2.65–1.88). They were also grouped into non-frail, pre-frail, and frail groups by KCL score cutoff points (non-frail: <3, pre-frail: 4–7, frail: >7) [33], and also grouped into non-depressive, moderate depressive, and depressive groups by HADS score cutoff points (non-depressive: <11, moderate depressive: 11–19, depressive: >19) [34]. In categorical analyses, differences in HADS or KCL scores between groups divided by WHO/QOL-26 scores were analyzed using Mann–Whitney U tests. Differences in KCL scores between groups divided by HADS score and differences in HADS scores between groups divided by KCL score were analyzed using Mann–Whitney U tests. In dimensional analyses, correlations of WHO/QOL-26 scores with HADS and KCL scores and correlations between HADS and KCL scores were analyzed using Spearman’s partial rank correlation tests with age as a covariate. Differences in volume of whole hippocampi or hippocampal subfields between groups divided by HADS or KCL scores were analyzed using Mann–Whitney U tests. There were significant differences in categorical analysis, these associations were confirmed using Spearman’s partial rank correlation tests with age and eTIV as covariates. To evaluate direct association between hippocampal volume and QOL, differences in volume of whole hippocampi or hippocampal subfields between groups divided by WHO/QOL-26 scores were analyzed using Mann–Whitney U tests. When significant differences were found in categorical analysis, these associations were confirmed using Spearman’s partial rank correlation tests with age and eTIV as covariates. Correlations between values of respiratory function (%VC, %FVC, %FEV1, %DLco/VA) and scores of WHO/QOL-26, HADS, KCL were analyzed using Spearman’s partial rank correlation tests with age as a covariate. Correlations between values of respiratory function (%VC, %FVC, %FEV1, %DLco/VA) and volume of whole hippocampi or hippocampal subfields were examined using Spearman’s partial rank correlation tests with age and eTIV as covariates. To evaluate direct and indirect relation, the factors which showed significant correlation were analyzed using step-wise multiple regression analysis. Statistical significance was set at *p* < 0.05 in all analyses.

## 3. Results

### 3.1. Association between QOL and Frailty and Depressive Symptoms

Subjects were divided into high QOL (*n* = 11), moderate QOL (*n* = 19), and low QOL (*n* = 10) groups (Table S1). HADS and KCL scores were significantly higher in the low QOL group compared with those in the high and moderate QOL groups (Table S1). In correlation analysis, WHO/QOL-26 scores were significantly negatively correlated with KCL (*r* = −0.596; *p* < 0.001) and HADS (*r* = −0.723; *p* < 0.001) scores (Table 2).

### 3.2. Association between Frailty and Depressive Symptoms

The patients were divided into non-frail (*n* = 9), pre-frail (*n* = 11), and frail (*n* = 20) groups (Table S2) and divided into non-depressive (*n* = 15), moderate depressive (*n* = 18), and depressive (*n* = 7) groups (Table S3). HADS scores were significantly higher in the frail group compared with the non-frail and pre-frail groups (Table S2). KCL scores were significantly higher in the depressive group compared with the non-depressive and moderate depressive groups (Table S3). In correlation analysis, there was significant correlation between HADS and KCL scores (*r* = 0.560; *p* < 0.001) (Table 2).

### 3.3. Association between Volume of Hippocampal Subfields and Frailty and Depressive Symptoms 

**Figure 1:**
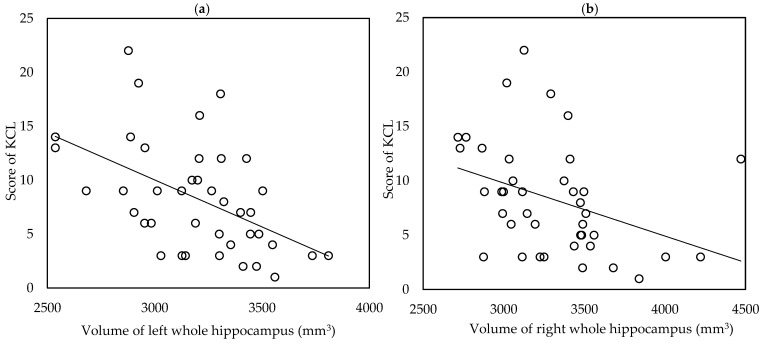
Scattergram for the association between KCL score and volume of whole hippocampus in the left (**a**) and right (**b**) hemispheres.

Volume of the left and right whole hippocampi, subiculum, and presubiculum were significantly lower in the frail group compared with non-frail groups (Table S4). Volume of the left and right whole hippocampi, left subiculum, and right presubiculum were significantly lower in the frail group compared with pre-frail groups (Table S4). None of the hippocampal subfield volumes had significant difference between groups divided by depressive symptoms (Table S5). In correlation analysis, KCL scores were significantly negatively correlated with volume of left and right whole hippocampi, subiculum and presubiculum (Table 3, Figure 1a,b).

### 3.4. Association between Hippocampal Subfield Volume and QOL

**Figure 2:**
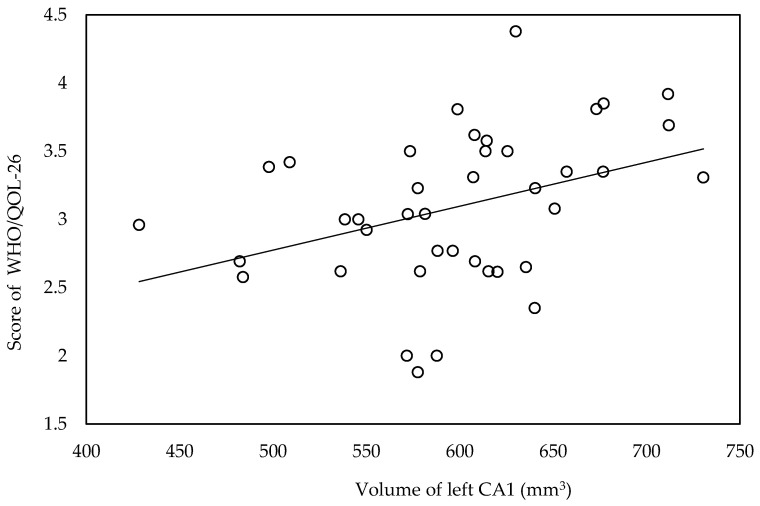
Scattergram for the association between WHO/QOL-26 score and volume of left CA1.

Volume of left CA1 was significantly lower in moderate and low QOL groups compared with high QOL group (Table S6). In correlation analysis, WHO/QOL-26 scores were significantly correlated with volume of left CA1 (*r* = 0.401, *p* = 0.013) (Figure 2). 

### 3.5. Association between Respiratory Values with QOL, Frailty, Depressive Symptoms and Hippocampal Subfield Volume

KCL scores were significantly correlated with values of %VC (*r* = −0.411, *p* = 0.009) and %FVC (*r* = −0.426, *p* = 0.007). There were no significant correlations between respiratory values and the volume of whole hippocampi and hippocampal subfields.

### 3.6. Step-Wise Multiple Regression Analyses for the Correlated Factors

**Figure 3:**
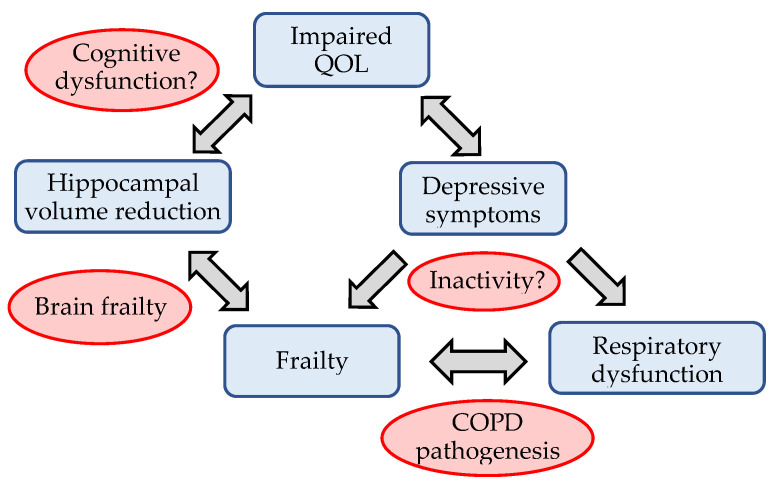
Association between QOL, frailty, depressive symptoms and hippocampal volume in patients with COPD. “Cognitive dysfunction” and “Inactivity” are presumption for future studies.

Step-wise multiple regression analyses revealed that WHO/QOL-26 score was sig-nificantly related to HADS score (β = −0.712, *p* < 0.001) and volume of left CA1 (β = 0.252, *p* = 0.017) (Table S7); HADS score was significantly related to WHO/QOL-26 score (β = −0.755, *p* < 0.001) (Table S7); KCL score was significantly related to HADS score (β = 0.565, *p* < 0.001), volume of left whole hippocampus (β = −0.394, *p* < 0.001) and value of %FVC (β = −0.353, *p* = 0.001) (Table S7); volume of left and right whole hippocampi, subiculum and presubiculum were significantly related to KCL scores (Table S8); volume of left CA1 was significantly related to WHO/QOL-26 score (β = 0.376, *p* = 0.017) (Table S8); value of %VC was significantly related with KCL score (β = −0.588, *p* = 0.003) and HADS score (β = 0.419, *p* = 0.029) (Table S9); value of %FVC was significantly related to KCL score (β = −0.607, *p* = 0.002) and HADS score (β = 0.411, *p* = 0.031) (Table S9). Associations revealed by step-wise multiple regression analyses are shown in Figure 3.

## 4. Discussion

This study confirmed association between poor QOL with frailty and with depressive symptoms. Depressive symptoms had impact on frailty and respiratory dysfunction. Interestingly, hippocampal volume was associated with frailty and QOL in patients with COPD, but not with depressive symptoms.

To our knowledge, this is the first study to show statistically significant direct association between volume reduction in the hippocampi and frailty, and therefore QOL in patients with COPD. It suggests a significant role of brain pathology in COPD prognosis. A high prevalence of frailty in patients with COPD (> 50%) was reported in a meta-analysis of an observational study [8] and in a national survey based in the United States [35]. Frailty is one of the worst prognostic factors related to increased rate of hospitalization [10], high mortality [10,11], greater disability [12,35], and to poor patient-reported outcomes and QOL [10,30]. Volume reduction in the hippocampi of patients with COPD has been reported [24,25], and relationships have been suggested between hippocampal volume loss in older adults with frailty [26] and with weakness and slowness [27]. Figure S1 shows the hippocampal subfield segmentation which was focused on in this study. Left and right subiculum and presubiculum volumes showed significant negative correlation with KCL scores. The subiculum and presubiculum are structures located within the hippocampus proper and entorhinal and other cortices, and are, therefore, a major output structure of the hippocampus [36,37,38]. Further research is needed to elucidate the relationship between frailty and reduced hippocampal volume and cognitive decline in patients with COPD.

Contrary to our expectations, the volume of hippocampal subfields did not have significant correlation with HADS scores. Among our patients with COPD, the number who scored above the “depressive” cutoff point in HADS was 7/40, while 20/40 scored above the “frail” cutoff point in KCL, suggesting lower severity of depressive symptoms than those of frailty. Moreover, while some studies have highlighted hippocampal volume reduction in patients with depression [39,40,41,42,43,44], depressive symptoms in COPD may have complex biological mechanisms arising from systemic hypoxia [45,46] and inflammation [45,46,47,48,49,50], contributing to the lack of statistically significant correlation between hippocampal volume and depressive symptoms in our patients with COPD.

While the volume of the whole hippocampi or of several hippocampal subfields were associated with frailty, the volume of the left CA1 was significantly associated solely with the QOL score. This suggests hippocampal volume had a weak association with QOL compared with frailty. Interestingly, CA1 was significantly associated with QOL, but not with frailty. CA1 is located between CA3 and the subiculum and modulate hippocampal circuitry [51,52], so further research is needed to elucidate any association between hippocampal circuit activity and QOL in patients with COPD.

Concerning the results of step-wise multiple regression analyses, our patients with COPD had a significant association between QOL and depressive symptoms and hippocampal volume reduction. Among our subjects, frailty was not directly associated with QOL, but it may impact upon QOL via reduction in hippocampal volume (brain frailty). Our results are in line with previous studies that reported a relationship between impaired QOL in patients with COPD and comorbid depression [13,16,53], as well as frailty [10]. QOL has been recognized as an important outcome in COPD because it is a predictor of subsequent exacerbations of COPD, hospitalization, and all-course mortality [2,54]. Association between depression and low physical activity has been reported in some cross-sectional studies [55,56], and a previous study with prospective design reported association between depressive symptoms and a reduction in physical activity six months later [57]. Bidirectional association between depression and clinical outcomes in COPD has been demonstrated in a meta-analysis of longitudinal studies [19]. In a longitudinal study of community-dwelling older adults, frailty was reported to predict persistent depressive symptoms [58]. Our results and those of these previous studies highlight the importance of intervention against frailty or depressive symptoms to improve QOL in patients with COPD.

Interestingly, in categorical analyses, patients in the low-QOL group showed worse frailty and depressive symptoms than patients in the moderate- and high-QOL groups, but there was no difference between high- and moderate-QOL groups. Similarly, patients in the frail group showed worse depressive symptoms than patients in the non-frail and pre-frail groups, but there was no difference between patients in the non-frail and pre-frail group. Patients in the depressive group showed worse frailty than patients in the non-depressive and moderate depressive groups, but there was no difference between patients in the non-depressive and moderate depressive groups. This suggests that early intervention during pre-frailty or moderate depressive symptoms could prevent the decline of QOL in patients with COPD. Our results also showed that frailty could be the cause of hippocampal volume reduction in patients with COPD. Previous studies have demonstrated reversibility of hippocampal volume reduction by increased physical activity [59,60]. This evidence suggests that early intervention for frailty may target brain frailty in patients with COPD, so assessment of the hippocampus, such as in our study could be useful for the management of COPD.

Our study has some limitations, and the results should be interpreted cautiously. First, healthy controls were not included in this study. While we found COPD pathogenic relation between respiratory dysfunction and frailty and their combined impacts upon hippocampal volume reduction and QOL, these associations were not compared with healthy controls. However, since healthy subjects usually do not have respiratory dysfunction, we have believed that our results are specific for COPD. Second, WHO/QOL-26 is not a QOL assessment tool specifically for patients with COPD. However, assessment by WHO/QOL has been widely used in disease groups, including for patients with COPD, in previous studies [61,62]. Third, treatment for COPD or several comorbidities with COPD might influence frailty, hippocampal volume, depressive symptoms and QOL. Notably, overlap with obstructive sleep apnea syndrome (OSAS) should be screened in future studies because some previous studies have reported association between OSAS and impaired QOL [63,64] and hippocampal volume reduction [65].

In conclusion, in this study there was significant association between frailty, depressive symptoms, and poor QOL. Hippocampal pathology could be related to frailty and to some extent with QOL in patients with COPD. Brain frailty accompanied by reduction in hippocampal volume is suggested to be a cause of poor QOL. Further research with a longitudinal design is needed to elucidate the causal relationship of our findings.

## Figures and Tables

**Table 1:** Characteristics of the subjects.

Measure	Mean	S.D.
Gender (male/female)	39/1	
Age (years)	70.63	8.21
BMI (kg/m^2^)	23.26	3.29
Smoking status (Cu/Ex/Non)	12/28/0	
Pack years	46.60	25.49
%VC (%)	97.00	18.67
%FVC (%)	98.70	19.26
%FEV_1_ (%)	73.96	18.07
%DLco/VA (%)	77.84	24.10
HADS	12.83	7.09
KCL	8.30	5.08
WHO/QOL-26	3.09	0.56

Abbreviations: S.D. = standard deviation, BMI = body mass index, Cu = current smoker, Ex = ex-smoker, Non = non-smoker, VC = vital capacity; FVC = forced vital capacity, FEV1 = forced expiratory volume in 1 s, DLco = diffusing capacity of lung carbon monoxide, VA = alveolar volume, HADS = Hospital Anxiety and Depression Scale, KCL = Kihon Checklist, WHO/QOL-26 = World Health Organization Quality of Life Assessment.

**Table 2:** Correlations among scores of WHO/QOL-26, KCL and HADS.

	WHO/QOL-26	KCL	HADS
WHO/QOL-26	1		
KCL	−0.596 *	1	
HADS	−0.723 *	0.560 *	1

Abbreviation: HADS = Hospital Anxiety and Depression Scale, KCL = Kihon Checklist, WHO/QOL-26 = World Health Organization Quality of Life Assessment. * *p* < 0.001.

**Table 3:** Correlations between volume of hippocampal subfields and KCL score.

	KCL	
	*r*	*p*
Left whole hippocampus	−0.458	0.004
Right whole hippocampus	−0.410	0.011
Left subiculum	−0.518	0.001
Left presubiculum	−0.401	0.013
Right subiculum	−0.497	0.001
Right presubiculum	−0.493	0.002

Abbreviation: KCL = Kihon Checklist.

## Data Availability

Research data are not shared.

## Supplementary Materials

The following are available online at https://www.mdpi.com/article/10.3390/biomedicines9091103/s1, Figure S1: Hippocampal subfield segmentation, Table S1: Differences of HADS or KCL scores between groups divided by WHO/QOL-26, Table S2: Differences of HADS scores between groups divided by KCL score, Table S3: Differences of KCL scores between groups divided by HADS score, Table S4: Differences in volume of whole hippocampi or hippocampal subfields between groups divided by KCL score, Table S5: Differences in volume of whole hippocampi or hippocampal subfields between groups divided by HADS score, Table S6: Differences in volume of whole hippocampi or hippocampal subfields between groups divided by WHO/QOL-26 score, Table S7: Step-wise multiple regression analysis of the relationship among WHO/QOL-26, KCL and HADS scores, volume of whole hippocampi or hippocampal subfields, and values of respiratory function, Table S8: Step-wise multiple regression analysis of the relationship between volume of whole hippocampi or hippocampal subfields with WHO/QOL-26, HADS and KCL scores and values of respiratory function, Table S9: Step-wise multiple regression analysis of the relationship between values of respiratory function with WHO/QOL-26, KCL and HADS scores and volume of whole hippocampi or hippocampal subfields.
