# Supplementary material for: Impact of Frailty on Hippocampal Volume in Patients with Chronic Obstructive Pulmonary Disease
Source: Biomedicines. 2021 Aug 28;9(9):1103. doi: 10.3390/biomedicines9091103 (PMC8468719; doi:10.3390/biomedicines9091103)
Supplement: Supplementary file 1 [file biomedicines-09-01103-s001.zip › biomedicines-1324402 supplementarytables.pdf]

Table S1. Differences of HADS or KCL scores between groups divided by WHO/QOL-26 score

	High QOL (n = 11)		Moderate QOL (n = 19)		Low QOL (n = 10)		High QOL vs Moderate QOL	High QOL vs Low QOL	Moderate QOL vs
	Mean	S.D.	Mean	S.D.	Mean	S.D.	*p*	*p*	*p*
WHO/QOL-26	3.74	0.26	3.08	0.24	2.39	0.31			
KCL	5.18	3.63	7.42	3.81	13.40	5.08	0.083	0.002 *	0.003 *
HADS	8.18	5.04	11.0	4.40	21.4	6.00	0.188	<0.001 *	<0.001 *

Abbreviation: WHO/QOL-26 = World Health Organization Quality of Life Assessment, QOL = quality of life, S.D. = standard deviation, KCL = Kihon Checklist, HADS = Hospital anxiety and depression scale. * $p < 0.05$

Table S2. Differences of HADS scores between groups divided by KCL score

	Non-frail (n = 9)		Pre-frail (n = 11)		Frail (n = 20)		Non-frail vs Pre-frail	Non-frail vs Frail	Pre-frail vs Frail
	Mean	S.D.	Mean	S.D.	Mean	S.D.	*p*	*p*	*p*
KCL	2.56	0.73	5.64	1.12	12.4	3.88			
HADS	8.00	5.43	9.82	3.34	16.7	7.30	0.517	0.005 *	0.007 *

Abbreviation: S.D. = standard deviation, KCL = Kihon Checklist, HADS = Hospital anxiety and depression scale. * $p < 0.05$

Table S3. Differences of KCL scores between groups divided by HADS score

	Non-depressive (n = 15)		Moderate depressinve (n = 18)		Depressive (n = 7)		Non-depressive vs Moderate depressive	Non-depressive vs Depressive	Moderate depressive vs
	Mean	S.D.	Mean	S.D.	Mean	S.D.	*p*	*p*	*p*
HADS	6.20	2.54	13.8	2.53	24.6	4.50			
KCL	6.00	3.72	8.11	4.69	13.7	5.06	0.197	0.002 *	0.025 *

Abbreviation: S.D. = standard deviation, KCL = Kihon Checklist, HADS = Hospital anxiety and depression scale. * $p < 0.05$

Table S4. Differences in volume of whole hippocampi or hippocampal subfields between groups divided by KCL score

	Non-frail (n = 9)		Pre-frail (n = 12)		Frail (n = 21)		Non-frail vs Pre-frail	Non-frail vs Frail	Pre-frail vs Frail
Volume (mm3)	Mean	S.D.	Mean	S.D.	Mean	S.D.	*p*	*p*	*p*
Left whole hippocampus	3398.72	273.49	3273.69	231.09	3065.50	278.49	0.305	0.016 *	0.039 *
Right whole hippocampus	3522.31	446.53	3352.33	212.59	3182.54	396.73	0.382	0.048 *	0.019 *
Left CA1	621.66	59.40	620.50	65.48	575.16	62.11	0.849	0.144	0.083
Left CA3	186.96	18.42	192.25	31.75	187.44	21.97	0.470	0.604	0.620
Left CA4	241.81	23.05	239.43	23.15	233.57	21.55	0.790	0.258	0.563
Left subiculum	462.28	55.33	435.34	37.09	394.75	52.39	0.342	0.008 *	0.015 *
Left presubiculum	341.97	34.32	307.97	42.44	289.01	43.66	0.053	0.006 *	0.342
Left parasubiculum	69.44	14.74	59.32	16.53	62.48	21.99	0.087	0.300	0.650
Left GC-ML-DG	275.03	30.07	268.67	25.76	263.36	26.80	0.382	0.172	0.620
Right CA1	653.52	103.27	631.12	36.50	614.29	90.69	0.676	0.322	0.127
Right CA3	207.89	24.33	205.69	20.81	204.75	31.43	0.470	0.346	0.509
Right CA4	259.42	28.82	256.71	19.28	248.85	36.90	0.732	0.109	0.091
Right subiculum	479.80	71.69	440.01	42.68	406.82	61.50	0.271	0.011 *	0.083
Right presubiculum	311.45	44.45	291.75	32.12	263.95	43.46	0.210	0.018 *	0.048 *
Right parasubiculum	60.43	15.43	58.33	14.36	53.85	14.72	0.790	0.322	0.457
Right GC-ML-DG	296.30	38.77	287.83	22.95	281.73	44.43	0.518	0.144	0.201

Abbreviation: S.D. = standard deviation, GC-ML-DG = granule cell and molecular layer of the dentate gyrus. * $p < 0.05$

Table S5. Differences in volume of whole hippocampi or hippocampal subfields between groups divided by HADS score

	Non-depressive (n = 15)		Moderate depressive (n = 18)		Depressive (n = 7)		Non-depressive vs Moderate depressive	Non-depressive vs Depressive	Moderate depressive vs Depressive
Volume (mm3)	Mean	S.D.	Mean	S.D.	Mean	S.D.	*p*	*p*	*p*
Left whole hippocampus	3235.03	334.26	3109.96	308.43	3321.55	263.84	0.219	0.916	0.304
Right whole hippocampus	3313.67	430.87	3205.25	306.85	3546.97	490.01	0.386	0.503	0.304
Left CA1	615.36	72.92	580.67	66.89	605.87	27.55	0.112	0.378	0.204
Left CA3	183.83	26.35	188.63	24.13	199.07	15.49	0.857	0.072	0.204
Left CA4	240.32	19.84	230.15	24.90	247.68	13.45	0.278	0.459	0.116
Left subiculum	443.63	53.33	403.70	55.96	417.60	50.96	0.120	0.217	0.952
Left presubiculum	322.30	50.24	292.08	42.44	307.65	35.70	0.120	0.698	0.545
Left parasubiculum	60.50	21.21	64.13	18.94	66.48	16.25	0.448	0.341	0.545
Left GC-ML-DG	271.81	25.52	258.18	29.21	281.91	15.59	0.138	0.597	0.053
Right CA1	640.14	77.89	603.53	67.85	663.46	115.20	0.406	0.751	0.333
Right CA3	203.63	15.32	201.66	25.31	220.62	44.37	0.691	0.503	0.397
Right CA4	256.07	21.92	244.22	24.57	271.21	52.05	0.206	0.698	0.397
Right subiculum	443.01	77.31	420.91	53.97	439.02	67.81	0.664	0.972	0.809
Right presubiculum	284.76	53.24	278.31	39.95	287.18	40.84	0.638	0.972	0.586
Right parasubiculum	55.13	13.27	57.36	16.18	57.60	15.45	0.638	0.698	0.952
Right GC-ML-DG	289.87	29.62	274.74	30.32	310.59	59.86	0.219	0.459	0.204

Abbreviation: S.D. = standard deviation, GC-ML-DG = granule cell and molecular layer of the dentate gyrus.

Table S6. Differences in volume of whole hippocampi or hippocampal subfields between groups divided by WHO/QOL-26

	High QOL (n = 11)		Moderate QOL (n = 19)		Low QOL (n = 10)		High QOL vs Moderate QOL	High QOL vs Low QOL	Moderate QOL vs Low QOL
Volume (mm3)	Mean	S.D.	Mean	S.D.	Mean	S.D.	*p*	*p*	*p*
Left whole hippocampus	3345.40	279.38	3149.51	295.73	3126.89	277.64	0.089	0.260	0.646
Right whole hippocampus	3442.38	448.96	3281.45	397.57	3201.34	258.38	0.333	0.260	0.582
Left CA1	639.78	46.46	580.98	73.31	584.73	47.88	0.017 *	0.049 *	0.927
Left CA3	195.51	22.02	185.23	25.72	187.61	22.75	0.378	0.622	0.680
Left CA4	242.86	20.00	234.82	22.17	234.83	24.89	0.237	0.324	0.714
Left subiculum	444.98	57.89	417.54	53.38	401.62	54.43	0.561	0.078	0.271
Left presubiculum	320.07	47.40	303.17	49.35	296.46	35.81	0.426	0.291	0.680
Left parasubiculum	61.07	19.51	63.41	21.48	65.05	14.86	0.914	0.526	0.521
Left GC-ML-DG	274.07	27.59	265.23	25.87	264.37	29.90	0.333	0.231	0.891
Right CA1	658.84	91.74	619.43	90.17	609.32	45.68	0.175	0.231	0.891
Right CA3	212.91	24.72	203.59	31.58	201.84	18.35	0.050	0.205	0.680
Right CA4	260.11	26.11	253.73	38.30	245.34	17.37	0.204	0.078	0.819
Right subiculum	455.52	79.88	430.59	58.21	410.29	57.27	0.561	0.181	0.383
Right presubiculum	288.69	49.48	284.03	43.94	271.92	42.89	0.813	0.481	0.335
Right parasubiculum	58.54	15.27	57.61	16.14	52.40	11.27	0.846	0.526	0.335
Right GC-ML-DG	296.44	35.62	285.64	45.17	277.94	23.30	0.081	0.105	0.927

Abbreviation: QOL = quality of life, S.D. = standard deviation, GC-ML-DG = granule cell and molecular layer of the dentate gyrus. * $p < 0.05$

Table S7. Step-wise multiple regression analysis of the relationship among WHO/QOL-26, KCL and HADS scores, volume of whole hippocampi or hippocampal subfields, and values of respiratory function.

	WHO/QOL-26			HADS score			KCL score		
	Standardized coefficient beta	t statistics	*p*	Standardized coefficient beta	t statistics	*p*	Standardized coefficient beta	t statistics	*p*
WHO/QOL-26				−0.755	−7.108	<0.001 *	−0.200	−1.350	0.186
HADS score	−0.712	−7.032	<0.001 *				0.565	5.844	<0.001 *
KCL	−0.147	−1.111	0.274	0.251	1.942	0.060			
Left whole hippocampus	−0.092	−0.571	0.571	0.049	0.442	0.661	−0.394	−4.066	<0.001 *
Right whole hippocampus	0.036	0.292	0.772	0.115	1.062	0.295	−0.063	−0.379	0.707
Left CA1	0.252	2.494	0.017 *	0.129	1.125	0.268	0.130	0.838	0.408
Left subiculum	−0.065	−0.526	0.602	−0.063	−0.559	0.579	−0.040	−0.208	0.837
Left presubiculum	−0.093	−0.880	0.385	−0.117	−1.092	0.282	0.021	0.159	0.874
Right subiculum	−0.007	−0.057	0.955	0.023	0.203	0.840	−0.161	−0.967	0.340
Right presubiculum	−0.044	−0.385	0.702	0.017	0.152	0.880	−0.141	−1.046	0.303
%VC	0.000	0.002	0.999	0.020	0.187	0.853	0.507	0.845	0.404
%FVC	0.010	0.096	0.924	0.015	0.143	0.887	−0.353	−3.698	0.001 *

Abbreviation: WHO/QOL-26 = World Health Organization Quality of Life Assessment, HADS = Hospital Anxiety and Depression Scale, KCL = Kihon Checklist, VC = vital capacity, FVC = forced vital capacity. * $p < 0.05$

Table S8. Step-wise multiple regression analysis of the relationship between volume of whole hippocampi or hippocampal subfields with WHO/QOL-26, HADS and KCL scores and values of respiratory function.

S8a	Left whole hippocampus			Left subiculum			Left presubiculum			Left CA1		
	Standardized coefficient beta	t statistics	*p*	Standardized coefficient beta	t statistics	*p*	Standardized coefficient beta	t statistics	*p*	Standardized coefficient beta	t statistics	*p*
WHO/QOL-26	−0.045	−0.252	0.803	−0.067	−0.391	0.698	−0.171	−0.924	0.361	0.376	2.501	0.017 *
HADS	0.238	1.349	0.186	0.101	0.584	0.563	0.055	0.294	0.771	0.257	1.125	0.268
KCL	−0.505	−3.610	0.001 *	−0.555	−4.115	<0.001 *	−0.425	−2.896	0.006 *	−0.178	−0.939	0.354
%VC	−0.116	−0.778	0.441	−0.034	−0.232	0.818	−0.032	−0.201	0.842	−0.014	−0.091	0.928
%FVC	−0.145	−0.968	0.339	−0.055	−0.374	0.710	−0.020	−0.123	0.903	−0.034	−0.221	0.826

Abbreviation:WHO/QOL-26 = World Health Organization Quality of Life Assessment, HADS = Hospital Anxiety and Depression Scale, KCL = Kihon Checklist, VC = vital capacity, FVC = forced vital capacity. * $p < 0.05$

S8b	Right whole hippocampus			Right subiculum			Right presubiculum		
	Standardized coefficient beta	t statistics	*p*	Standardized coefficient beta	t statistics	*p*	Standardized coefficient beta	t statistics	*p*
WHO/QOL-26	−0.043	−0.226	0.822	−0.069	−0.385	0.703	−0.203	−1.117	0.271
HADS	0.307	1.634	0.111	0.214	1.203	0.236	0.298	1.652	0.107
KCL	−0.370	−2.452	0.019 *	−0.497	−3.526	0.001 *	−0.449	−3.102	0.004 *
%VC	−0.092	−0.572	0.571	−0.123	−0.820	0.417	−0.031	−0.202	0.841
%FVC	−0.119	−0.733	0.468	−0.125	−0.828	0.413	−0.020	−0.126	0.900

Abbreviation: WHO/QOL-26 = World Health Organization Quality of Life Assessment, HADS = Hospital Anxiety and Depression Scale, KCL = Kihon Checklist, VC = vital capacity, FVC = forced vital capacity. * $p < 0.05$

Table S9. Step-wise multiple regression analysis of the relationship between values of respiratory function with WHO/QOL-26, KCL and HADS scores and volume of whole hippocampi or hippocampal subfields.

	%VC			%FVC		
	Standardized coefficient beta	t statistics	*p*	Standardized coefficient beta	t statistics	*p*
WHO/QOL-26	−0.223	−0.966	0.340	−0.216	−0.943	0.352
HADS	0.419	2.266	0.029 *	0.411	2.237	0.031 *
KCL	−0.588	−3.176	0.003 *	−0.607	−3.309	0.002 *
Left whole hippocampus	−0.232	−1.362	0.182	−0.264	−1.570	0.125
Right whole hippocampus	−0.201	−1.247	0.220	−0.226	−1.424	0.163
Left CA1	−0.181	−1.172	0.249	−0.203	−1.338	0.189
Left subiculum	−0.082	−0.461	0.648	−0.107	−0.610	0.546
Left presubiculum	−0.052	−0.320	0.751	−0.038	−0.236	0.815
Right subiculum	−0.228	−1.347	0.186	−0.266	−1.348	0.186
Right presubiculum	−0.141	−0.835	0.409	−0.125	−0.742	0.463

Abbreviation: WHO/QOL-26 = World Health Organization Quality of Life Assessment, HADS = Hospital Anxiety and Depression Scale, KCL = Kihon Checklist, VC = vital capacity, FVC = forced vital capacity. * $p < 0.05$
